# 

**Published:** 1918-07-13

**Authors:** 


RATES OF SUBSCRIPTION: Twelve months, 10/10; Six months, 5/5; three months, 2/9, post free to any address in the United Kingdom. Abroad, Twelve months, 13/-; Six months, 6/6; Three months, 3/3 post free. **THE HOSPITAL "Index" to Volume LXIII., October 1917 to March 1918, is now ready, price 3½d. per copy, post free.** Address The Manager, THE HOSPITAL, "The Hospital" Building, 28/29 Southampton Street, Strand, London, W.C. 2.

To obtain **The HOSPITAL**
regularly **EVERY THURSDAY** a definite order **must** be placed with your Newsagent **at once.** All Newsagents, Booksellers, and Railway Bookstalls will gladly book your order, but copies cannot be stocked for sale to chance customers.

### The Segregation of Consumptives in London.

THE Metropolitan Boroughs Standing Joint Committee is asking the Local Government Board and the Metropolitan Asylums Board to place an institution at the disposal of local authorities for the accommodation and isolation of advanced cases of consumption.

### The Nurses' New Charter of Liberty.

"IERNE'S" invitation to the nurses to become articulate in support of this Charter has moved all nurses already. Matrons, sisters, nurses, and probationers are sending their postcards in increasing numbers, setting out what each regards as a reasonable and proper allowance of off-time and holidays, adding at the foot, not for publication, the name and address of the writer, her rating in nursing, with the names of her fellow-nurses who share her views. We invite all nurses to follow this advance guard by filling up postcards, posting them at once to "Ierne," c/o THE HOSPITAL, 28 and 29 Southampton Street, Strand, London, W.C. 2, and to recruit their friends too.

